# Cigarette Smoking, Birthweight and Osteoporosis in Adulthood: Results from the Hertfordshire Cohort Study

**DOI:** 10.2174/1874312900802010033

**Published:** 2008-05-06

**Authors:** M.M Moinuddin, K.A Jameson, H.E Syddall, A. Aihie Sayer, H.J Martin, S Robinson, C Cooper, E.M Dennison

**Affiliations:** MRC Resource Centre, University of Southampton, Southampton General Hospital, Southampton SO16 6YD, UK

## Abstract

We looked for interaction between early environment and adult lifestyle in determination of bone mineral content (BMC) and bone mineral density (BMD) among 498 men and 468 women for whom birth records were available. Participants completed a health questionnaire, and bone densitometry (DXA) of the lumbar spine and femoral neck performed.

We found no relationships between cigarette and alcohol consumption, physical activity and either BMC or BMD after adjustment for age, body mass index, dietary calcium, social class, HRT use and years since menopause. However, male current smokers in the lowest third of birth weight had lower femoral neck BMD than ex- or never smokers from the lowest birth weight third (p value for interaction term = 0.04). Similar trends were seen with femoral neck BMC and lumber spine BMC.Individuals of lower birth weight may be particularly vulnerable to the effects of bone noxious stimuli such as cigarette smoking.

## INTRODUCTION

Epidemiological studies have shown that individuals of low birthweight may be at increased risk of osteoporosis in adult life [1-6]. Lifestyle factors in adulthood also affect bone mineral content (BMC) and bone mineral density (BMD) although the extent of this influence may vary between individuals; previous studies have suggested that the early environment may interact with adult lifestyle to determine risk factors for common diseases. In one recent study by Robinson *et al*. individuals of low birth weight were particularly susceptible to the effect of high dietary fat intakes when blood cholesterol levels were measured [7]. To investigate whether a similar interaction might operate between later lifestyle factors and growth in early life to determine the risk of adult osteoporosis, we examined the relationships between birth weight, lifestyle factors and adult BMD and BMC in a cohort of men and women from Hertfordshire, UK.

## MATERIALS AND METHODS

Four hundred and ninety eight men and 468 women aged 59-71 years were recruited to this study, which was designed to examine the relationship between growth in infancy and the subsequent risk of osteoporosis. The selection procedure for these individuals was as follows: in brief, with the help of the National Health Service Central Registry at Southport, and Hertfordshire Family Health Service Association, we traced men and women who were born during 1931-39 in Hertfordshire, and still lived there during the period 1998-2003. After obtaining written permission from each subject’s General Practitioner, we approached each person by letter, to ask them if they would be willing to be contacted by one of our research nurses. If they agreed, a research nurse performed a home visit, where they administered a structured questionnaire. This included information on socioeconomic status, medical history, cigarette smoking, alcohol consumption and dietary calcium intake. Physical activity was assessed by a previously validated questionnaire [8]. The subject was then invited to attend a local clinic.

Height was measured to the nearest 0.1cm using a Harpenden pocket stadiometer (Chasmors Ltd., London, UK) and weight to the nearest 0.1kg on a SECA floor scale (Chasmors Ltd., London, UK). Body mass index (BMI) was calculated as weight divided by height 2 (kg/m^2^).

Bone mineral density and bone mineral content were measured in each subject, by dual energy X-ray absorptiometry at the lumbar spine and proximal femur (neck, total, intertrochanteric and trochanteric regions, Wards triangle) using a Hologic QDR 4500 instrument. Measurement precision error, expressed as coefficient of variation, was 1.55% for lumbar spine BMD, 1.45% for total femur and 1.83% for femoral neck BMD for the Hologic QDR 4500; these figures were obtained by twenty five volunteers who were not part of the study undergoing two scans on the same day, getting on and off the table between examinations. Short-term (two month) precision error for the QDR 4500 was less than 1% for both sites (manufacturers figures). Individuals taking drugs known to alter bone metabolism (such as bisphosphonates) were excluded from this part of the study, although women taking hormone replacement therapy (HRT) were allowed to participate, due to the large numbers of women taking this medication (with appropriate adjustment in all analyses). There were no other exclusion criteria to this part of the study, and subjects were approached for consent as they attended clinic.

Variables were summarised with the use of means and standard deviations (SD). Multiple linear regression was used to explore the main and interactive effects of birthweight and lifestyle on BMD and BMC. Continous measures of birth weight were used in these analyses, though results were summarised by thirds of birthweight for ease of presentation. The linear regression models were performed both unadjusted and adjusted for age, BMI, dietary calcium, social class, and HRT use and years since menopause in women. All analyses were carried out using STATA (statistical package version 10)

Ethical permission for the study was granted by the East and North Hertfordshire Ethical Committees. All participants gave written informed consent.

## RESULTS

The mean age of the men and women studied was 64.8 and 66.3 years respectively. The weekly alcohol consumption, physical activity, current BMI, socioeconomic status, BMD and BMC of the men and women studied are shown in Table **1**. Thirty four percent of the men and 62% of the women had never smoked, while 52% of the men and 28% of the women were ex-smokers. The remaining 15% of the men and 10% of the women were current smokers. Four percent of men and 18% of women were non-drinkers, while 21% of the men and 12% of the women were moderate drinkers (i.e., 11-21 units per week for men, 8-14 units per week for women). Twenty five percent of men and 3% of women consumed greater than the recommended units of alcohol per week (i.e., > 21 units per week for men, >14 units per week for women). The geometric mean daily calcium intake was 1219 mg in men and 1085 mg in women.

We found no relationship between the lifestyle factors studied (alcohol and cigarette consumption, physical activity) and either BMD or BMC in this group as a whole (Table **2**). However, when we examined the associations between BMD or BMC and lifestyle factors in men and women according to tertile of birth weight, smoking status and birth weight appeared to interact in the determination of femoral neck and total femoral bone mineral content for men (Table **3**). Hence, in the lowest tertile of birth weight, current male smokers had a mean femoral neck BMC of 4.49 (SD 0.55) gcompared with a mean femoral neck BMC of 4.87 (SD 0.75) g for ex-smokers and a mean femoral neck BMC of 4.97 (SD 0.79) g for never smoked (*p* for interaction = 0.01 for current smoking and birth weight in men). This relationship was attenuated by adjustment for age, BMI, dietary calcium and social class (p=0.07). Similarly, in the lowest third of birth weight, current male smokers had a mean femoral neck BMD of 0.77 (SD 0.09) g/cm^2 ^compared with a mean femoral neck BMD of 0.85 (SD 0.12) g/cm^2^ for ex-smokers and a mean femoral neck BMD of 0.84 (SD 0.12) g/cm^2^ for never smoked (*p* for interaction = 0.04 for current smoking and birth weight in men). Again, this relationship was attenuated after adjustment for age, BMI, dietary calcium and social class (p=0.18) (Table **4**). A similar trend was seen in the relationship between lumbar spine BMC and smoking and birth weight in men, but this was not significant. If only smoking and birth weight were included in the multivariate regression model, the adjusted R^2 ^ranged from 0.3% to 2.1%, with an adjusted R^2 ^value for total femoral BMC in men of 2.1%, reflecting the inherent biological variability and both measured and unmeasured confounders in epidemiological studies. An estimate of lifetime total cigarette consumption (cigarette pack years) was not significantly associated with BMC or BMD at any site.

The interaction between BMD or BMC and smoking and birth weight was not seen in women, and nor were any interactions observed between early life and alcohol consumption or physical activity levels as determinants of BMC or BMD.

## DISCUSSION

We have found an interaction between smoking status and birth weight in a cohort of men from Hertfordshire. In males of low birth weight, smoking was found to be associated with a low BMD. Our findings suggest that individuals of lower birth weight may be particularly vulnerable to the effects of noxious stimuli to the skeleton, such as cigarette smoking. We have not found a similar interaction in women. Although this may reflect a true lack of effect in women, we suspect that our findings reflect limited power to detect a similar association in women (45 (10%) of the women were current smokers while 290 (62%) women had never smoked; in men 73 (15%) were current smokers and 167 (34%) had never smoked).

Our study has a number of limitations. The individuals recruited were selected because they had been born in Hertfordshire, and continued to live there at the age of 60-75 years, as in previous studies. However, we have previously demonstrated that the Hertfordshire populations studied have similar smoking characteristics and bone density to national figures, suggesting that selection bias is minimal [9]. Also, as discussed above, the lack of interaction in women could either reflect a true lack of interaction or limited study power due to low numbers of female current smokers and limited sample size in general.

An inverse relationship between smoking and bone density is well established [10], and is due to multiple factors including an earlier menopause in females, reduced body weight and enhanced metabolic breakdown of exogenous oestrogens (again in women). *In vitro*, cigarette smoke extract inhibited *in vitro *differentiation of osteoprogenitor cells to osteoblast like cells. In a recent meta-analysis [11], the results of 48 published studies were combined. The authors concluded that although they were able to demonstrate no significant difference in bone density at age 50 between smokers and non-smokers, bone density in women diminished by about an extra 2% for every 10 year increase in age, with a difference of 6% at age 80. Although the confounding effects of body mass index and oestrogen were mentioned, this meta-analysis could not fully address the lifestyle differences between smokers and non-smokers. Cigarette smoking was recently the subject of a further recent meta-analysis that found current smoking was associated with a significantly increased risk of any fracture compared to non-smokers [12]. Adjustment for BMD had little impact on the increased risk. Risk ratios were significantly higher in men than women for all fractures and for osteoporotic fractures, but not for hip fracture. It has been suggested that this may reflect a dose response effect, and the higher consumption of tobacco among males. Low BMD accounted for only 23% of the smoking-related risk of hip fracture, while adjustment for BMI had a small downward effect on risk for all fracture outcomes. A smoking history was associated with a significantly increased risk of fracture compared with individuals without a smoking history, but the risk was lower than for current smokers.

Lorentzon *et al*. recently performed a study of areal and volumetric BMD among young male Scandinavian smokers [13]. Despite a low daily intake (average 10 cigarettes per day) and a reasonably short duration of smoking (average 4 years), they were able to demonstrate significantly lower a real BMD, and lower tibial trabecular volumetric BMD among the 9% of the study population who smoked daily. Hence this study would suggest an effect of smoking on peak bone mass in addition to the previous reports of an effect on age related bone loss [14, 15]. While the exact mechanism of these effects is unclear, it is likely to include an effect on sex hormones (female smokers have a premature menopause; male smokers have higher levels of testosterone, and both sexes have increased ACTH secretion and adrenal steroids), lower levels of circulating 25(OH)D, and a number of lifestyle variables associated with smoking may contribute to the observed effects, including lower BMI and levels of physical activity. At a cellular level, both stimulation and inhibition of osteoblast formation and activity have been reported, while in other cell types tobacco has been reported to induce apoptosis [16].

Other studies have established that responses to adverse influences in adult life are conditioned by early growth. In the American nurses' health study, the highest risk of coronary heart disease was found among women who had low birth weight and high BMI as adults [17]. Most recently, our group has suggested that responses to dietary fat are also conditioned by early growth, which could help to explain the inconsistent associations observed between birth weight and serum cholesterol concentration [7]. These findings, like those in the study reported here, are consistent with the ideas of Dubos who wrote that 'the effects of the physical and social environments cannot be understood without knowledge of individual history' [18].

In conclusion, we have found that current smoking was associated with a low BMD in males of low birth weight. If replicated, our findings could have implications for public health. Therefore male smokers of lower birth weight would define a group who are at increased risk of osteoporosis and who might benefit most from a cessation of smoking.

## Figures and Tables

**Table 1.** Characteristics of Study Participants

Characteristics	Males (n=498)	Females (n=468)
Age (yrs)	64.8 (2.5)	66.3 (2.6)
BMI (kg/m^2^)^1^	26.6 (1.1)	26.9 (1.2)
Alcohol consumption (units per week)^2^	10.0 (3.0, 22.5)	2.5 (0.5, 7.0)
Habitual Activity (%)^3^	64.0 (14.8)	61.3 (14.9)
N (%) Current smokers	73 (14.7)	45 (9.6)
N (%) Ex-smokers	258 (51.8)	132 (28.2)
N (%) Never smoked	167 (33.5)	290 (62.0)
N (%) Current manual social class IIIM-V^4^	277 (55.6)	286 (61.1)
N (%) Current non-manual social class I-IIINM^4^	193 (38.8)	182 (38.9)
Femoral Neck BMD (g/cm^2^)^5^	0.85 (0.12)	0.76 (0.12)
Total femoral BMD (g/cm^2^)^5^	1.04 (0.13)	0.90 (0.13)
Lumbar Spine BMD (g/cm^2^)^6^	1.08 (0.16)	0.96 (0.17)
Femoral Neck BMC (g)^5^	5.0 (0.8)	3.9 (0.7)
Total femoral BMC (g)^5^	48.4 (8.1)	32.5 (5.8)
Lumbar spine BMC (g)^7^	77.4 (15.6)	57.2 (13.2)

Mean (SD) unless stated otherwise.

1 Geometric mean and SD.

2 Median and IQR among drinkers. 20 men and 86 women stated that they do not drink alcohol.

3 Standardised score ranging 0-100 derived from frequency of gardening, housework, climbing stairs and carrying loads in a typical week. Higher scores indicate greater level of activity.

4 Social class was unclassified for 28 men.

5 Data on femoral neck BMD and BMC and total femoral BMD and BMC was missing for 3 men and 1 woman.

6 Data on lumbar spine BMD was missing for 1 man.

7 Data on lumbar spine BMC was missing for 3 men and 25 women.

**Table 2.** Univariate Analyses of Smoker Status, Alcohol Consumption and Physical Activity as Explanatory Variables for Femoral Neck BMD and BMC in Men

		Femoral Neck BMD	
		Regression Coefficient (95% CI)	p-Value
Smoker status:	Never smoked	-	-
	Ex-smoker	0.005 (-0.019, 0.029)	0.67
	Current smoker	-0.004 (-0.038, 0.029)	0.80
Alcohol consumption (units/week)		0.0005 (-0.0002, 0.0012)	0.17
Physical activity		0.0001 (-0.0006, 0.0008)	0.77
		**Femoral Neck BMC**	
		**Regression Coefficient (95% CI)**	**p-Value**
Smoker status:	Never smoked	-	-
	Ex-smoker	-0.03 (-0.18, 0.13)	0.74
	Current smoker	-0.02 (-0.24, 0.20)	0.83
Alcohol consumption (units/week)		0.004 (-0.001, 0.008)	0.12
Physical activity		0.000 (-0.004, 0.005)	0.87

**Table 3.** Bone Mineral Content (BMC) According to Birth Weight

	Men			Women		
	Lowest Third Birth Weight	Middle Third Birth Weight	Highest Third Birth Weight	Lowest Third Birth Weight	Middle Third Birth Weight	Highest Third Birth Weight
**Femoral Neck BMC**						
Current smoker	4.49 (0.55)	5.20 (1.03)	5.26 (0.77)	3.89 (0.78)	4.04 (0.70)	3.96 (0.65)
	n = 23	n = 24	n = 26	n = 15	n = 17	n = 13
Ex-smoker	4.87 (0.75)	5.00 (0.80)	5.11 (0.78)	3.92 (0.64)	4.06 (0.71)	3.98 (0.72)
	n = 83	n = 81	n = 91	n = 50	n = 42	n = 39
Never smoked	4.97 (0.79)	5.05 (0.86)	5.03 (0.67)	3.75 (0.61)	3.89 (0.73)	3.90 (0.61)
	n = 51	n = 67	n = 49	n = 130	n = 100	n = 60
	*p = 0.01 (p = 0.07 adjusted)*			*p = 0.19 (p = 0.29 adjusted)*		
**Total Femoral BMC**						
Current smoker	44.7 (7.3)	50.0 (8.7)	51.8 (9.7)	31.6 (4.3)	33.0 (6.2)	35.3 (7.7)
	n = 23	n = 24	n = 26	n = 15	n = 17	n = 13
Ex-smoker	46.3 (8.6)	48.9 (8.0)	49.2 (8.0)	32.4 (5.9)	33.8 (6.2)	32.9 (5.1)
	n = 83	n = 81	n = 91	n = 50	n = 42	n = 39
Never smoked	48.4 (8.5)	48.8 (7.3)	48.4 (8.0)	31.4 (5.8)	32.2 (6.0)	33.5 (5.4)
	n = 51	n = 67	n = 91	n = 130	n = 100	n = 60
	*p = 0.01 (p = 0.06 adjusted)*			*p = 0.98 (p = 0.69 adjusted)*		
**Lumbar Spine BMC**						
Current smoker	73.1 (11.4)	79.2 (12.9)	75.2 (13.3)	57.9 (8.1)	58.6 (12.1)	63.3 (21.9)
	n = 23	n = 24	n = 25	n = 15	n = 17	n = 10
Ex-smoker	74.5 (15.2)	78.5 (14.9)	80.9 (17.0)	57.9 (13.2)	60.1 (12.0)	57.4 (15.5)
	n = 84	n = 81	n = 92	n = 49	n = 40	n = 39
Never smoked	75.8 (16.3)	79.3 (15.7)	75.3 (16.9)	54.0 (12.5)	57.5 (14.1)	58.7 (11.3)
	n = 50	n = 67	n = 49	n = 121	n = 95	n = 57
	*p = 0.69 (p = 0.96 adjusted)*			*p = 0.98 (p = 0.98 adjusted)*		

Figures given are mean BMC (g) with standard deviations in parentheses. P-values given are for the interaction between current smoker and birth weight, with and without adjusting for age, BMI, dietary calcium, social class and HRT use and years since menopause in women.

**Table 4.** Femoral Neck BMD According to Birth Weight in Men

	Lowest Third Birth Weight	Middle Third Birth Weight	Highest Third Birth Weight
Current smoker	0.77 (0.09)	0.89 (0.14)	0.87 (0.13)
	n = 23	n = 24	n = 26
Ex-smoker	0.85 (0.12)	0.86 (0.12)	0.86 (0.12)
	n = 83	n = 81	n = 91
Never smoked	0.84 (0.12)	0.85 (0.13)	0.85 (0.10)
	n = 51	n = 67	n = 49
	*p = 0.04 (p = 0.18 adjusted)*		

Figures given are mean femoral neck BMD (g/cm^2^) with standard deviations in parentheses. The p-values given are for the interaction between current smoker and birth weight, with and without adjusting for age, BMI, dietary calcium and social class.
